# A retrospective genomic analysis of drug-resistant strains of *M*. *tuberculosis* in a high-burden setting, with an emphasis on comparative diagnostics and reactivation and reinfection status

**DOI:** 10.1186/s12879-019-4739-z

**Published:** 2020-01-07

**Authors:** Kurt Wollenberg, Michael Harris, Andrei Gabrielian, Nelly Ciobanu, Dumitru Chesov, Alyssa Long, Jessica Taaffe, Darrell Hurt, Alex Rosenthal, Michael Tartakovsky, Valeriu Crudu

**Affiliations:** 1 Office of Cyber Infrastructure & Computational Biology, National Institute of Allergy and Infectious Diseases, National Institutes of Health, Bethesda, MD USA; 2 Microbiology and Morphology Laboratory, Institute of Phthisiopneumology, Chisnau, Moldova; 3 Department of Pneumology and Allergology, Nicolae Testemitanu State University of Medicine and Pharmacy, Chisinau, Moldova; 4 Division of Clinical Infectious Diseases, Research Center Borstel, Leibniz Lung Center, Borstel, Germany

**Keywords:** *Mycobacterium tuberculosis*, Recurrent infection, Multi-drug resistant, Extensively-drug resistant, Moldova

## Abstract

**Background:**

Recurrence of drug-resistant tuberculosis (DR-TB) after treatment occurs through relapse of the initial infection or reinfection by a new drug-resistant strain. Outbreaks of DR-TB in high burden regions present unique challenges in determining recurrence status for effective disease management and treatment. In the Republic of Moldova the burden of DR-TB is exceptionally high, with many cases presenting as recurrent.

**Methods:**

We performed a retrospective analysis of *Mycobacterium tuberculosis* from Moldova to better understand the genomic basis of drug resistance and its effect on the determination of recurrence status in a high DR-burden environment. To do this we analyzed genomes from 278 isolates collected from 189 patients, including 87 patients with longitudinal samples. These pathogen genomes were sequenced using Illumina technology, and SNP panels were generated for each sample for use in phylogenetic and network analysis. Discordance between genomic resistance profiles and clinical drug-resistance test results was examined in detail to assess the possibility of mixed infection.

**Results:**

There were clusters of multiple patients with 10 or fewer differences among DR-TB samples, which is evidence of person-to-person transmission of DR-TB. Analysis of longitudinally collected isolates revealed that many infections exhibited little change over time, though 35 patients demonstrated reinfection by divergent (number of differences > 10) lineages. Additionally, several same-lineage sample pairs were found to be more divergent than expected for a relapsed infection. Network analysis of the H3/4.2.1 clade found very close relationships among 61 of these samples, making differentiation of reactivation and reinfection difficult. There was discordance between genomic profile and clinical drug sensitivity test results in twelve samples, and four of these had low level (but not statistically significant) variation at DR SNPs suggesting low-level mixed infections.

**Conclusions:**

Whole-genome sequencing provided a detailed view of the genealogical structure of the DR-TB epidemic in Moldova, showing that reinfection may be more prevalent than currently recognized. We also found increased evidence of mixed infection, which could be more robustly characterized with deeper levels of genomic sequencing.

## Background

While global rates of incidence of tuberculosis (TB) have been falling at an average of 2% per year between 2000 and 2014, the incidence of drug resistant (DR) TB remains steady [1]. Many Eastern European countries have high rates of multidrug-resistant (MDR) and extensively drug-resistant (XDR) tuberculosis and are included among the WHO High-burden countries lists [1]. MDR tuberculosis is defined as infections resistant to the first-line antibiotics rifampicin and isoniazid. XDR tuberculosis is defined as an infection that is MDR and additionally resistant to at least one drug in both of the two classes used to treat MDR: fluoroquinolones and the second-line injectable drugs (amikacin, capreomycin or kanamycin).

High burden and poor clinical or public health control of TB may also influence the nature of recurrent TB cases. Recurrent TB, defined as TB that occurs after a patient has been considered cured by standard TB treatment, may arise due to exogenous reinfection or endogenous reactivation [2]. Endogenous reactivation could be due to ineffective TB treatment and/or the emergence of drug-resistance within an individual [2, 3]. Reinfection from another individual is an example of exogenous reinfection. Recurrent TB cases are reported as “relapse” cases (i.e., retreatment cases after treatment success) by WHO standard definitions, which does not distinguish true relapses from reinfection cases. Understanding the extent to which either scenario (reinfection or reactivation/relapse) is occurring in recurrent TB infections allows for application of appropriate control measures.

Molecular approaches can help differentiate between reactivation or reinfection among recurrent TB cases [4, 5]. In combination with epidemiological, clinical, and laboratory data, they can also reveal insight into transmission dynamics of TB, including DR-TB. Whole genome sequencing of *Mycobacterium tuberculosis (Mtb)* isolates has an advantage over genotyping in that it can evaluate variation across the entire genome. It also provides a more comprehensive and precise analysis of phylogenetic relationships and transmission dynamics that can help determine whether recurrent TB cases are due to reinfection or reactivation.

The Republic of Moldova is a small Eastern European country with a high MDR-TB burden [1]. National drug resistance surveys between 2006 and 2017 showed that MDR-TB prevalence among new cases increased from 5.0% in 2000 to 26% in 2017, and among previously treated patients, increased from 33.2% up to 56% (all data from the National TB registry of Moldova) [6, 7]. Various economic and cultural reasons contribute to this situation [8–10] which is exacerbated by long-term hospitalization and inadequate infection control measures in these facilities [11].

To understand the genomic basis of DR-TB in Moldova, we carried out whole-genome sequencing of *Mtb* isolates from 190 MDR (non-XDR), XDR, and drug-sensitive TB patients, identified and collected as part of the TB Portals Program [12]. Paired longitudinal samples from 87 of these patients with recurrent cases were used to characterize the nature of *Mtb* reactivation or reinfection in these cases.

## Methods

### Sample acquisition and selection

All isolates submitted for complete genome sequencing were selected from the biobank of *Mycobacterium tuberculosis* from the National TB Reference Laboratory (NRL) at the Institute of Phthisiopneumology in Chisinau, Moldova. This biobank contains more than 40,000 isolates, collected beginning in 2007 from TB patients who signed an informed consent agreement. Isolates were selected randomly from available data in the NRL and the National TB Register (http://simetb.ifp.md/SimeTB.ViewDB/default.asp), while attempting to provide an even distribution of DR and susceptible cases. Longitudinal samples were taken from patients with recurrent cases who have a minimum of two samples with DST results.

These samples were also collected as part of participation in the TB Portals Program. Their associated metadata, including clinical and bacterial genomic information and radiologic images, are publicly available at: https://data.tbportals.niaid.nih.gov.

### Culture isolation and drug susceptibility testing

Samples were cultured and subjected to DST when they were received at the Institute of Phthisiopneumology in Chisinau, Moldova. The smears for direct microscopy were prepared by Ziehl-Neelsen stain to detect acid-fast bacilli. The sputum samples were processed by using N-acetyl-L-cysteine and sodium hydroxide (NALC/NaOH). *M. tuberculosis* isolation was performed by culturing samples on solid media (Lowenstein-Jensen) and the MGIT system liquid media according to the instructions supplied by the MGIT system manufacturer (Becton Dickinson, Sparks, MD, USA).

Susceptibility testing with the automated MGIT system was performed with liquid cultures that tested positive at least 1 day but no more than 2 days earlier by following the manufacturer’s instructions using the SIRE drug kit [13]. The lyophilized antibiotics were reconstituted in distilled water and added to MGIT tubes supplemented with 0.8 ml of the enrichment solution (BACTEC MGIT SIRE supplement; Becton Dickinson). The DST assays were performed with the following final drug concentrations: 0.1 mg/L for Isoniazid, 1.0 mg/L for rifampicin, 1.0 mg/L for streptomycin and 5.0 mg/L for ethambutol. All of the drug-containing tubes were inoculated with 0.5 ml of MGIT culture. A SIRE drug-free control was also inoculated with 0.5 ml of a 1:100 dilution of the positive culture broth in sterile saline. The tubes were placed in the MGIT rack, incubated in the cabinet drawer of the MGIT system and were continuously monitored. The results indicating susceptibility or resistance were interpreted and reported automatically by the MGIT system using predefined algorithms that compare bacterial growth in the drug-containing tube with the growth in the drug-free control tube. For second line TB drugs the following concentrations (all mg/L) were used: amikacin-30.0, kanamycin-30.0, capreomycin-40, moxifloxacin-2.0, levofloxacin-2.0, ofloxacin-4.0, ethionamid-40.

PCR-based line probe assays (LPAs) were performed to directly test for the presence of mutations associated with drug resistance in the isolates. GenoType MTBDRplus and GenoType MTBDRsl (Hain Life Sciences, Nehren, Germany) were used to test *Mtb* isolates. Testing was performed according to the manufacturer’s recommendations. GeneXpert MTB/RIF (Cepheid, CA, USA) real-time PCR test was performed directly on clinical samples without prior extraction according to the manufacturer’s instructions.

### DNA extraction and sequencing

Among the samples selected for DNA extraction were pairs of longitudinal samples from patients who had been treated but suffered a recurrence of symptoms requiring a second course of treatment. DNA was extracted from cultures grown on Lowenstein-Jensen slants using the CTAB protocol [14]. Isolates were sequenced on Illumina platforms using a paired-end library design. Sequence reads were mapped to the *Mtb* reference sequence H37Rv NC_018143 using the BWA aligner (version 0.7.17-r1188) [15]. Variants were called using the Pilon software package (version 1.21) [16] and then annotated with the SNPeff package (version 4.3 k) [17]. All isolates in this study meet the minimum quality threshold of having at least 95% of the reference TB genome covered at 10x or greater and at least 80% of the total reads from the isolate mapped to the TB reference genome. The TB profiler software version 2.6 [18] was used to identify patients with low level DR infections and to determine the numeric SNP barcode [19] lineage designations. Digital spoligotypes were calculated using the lorikeet software [20].

The presence of SNPs associated with multiple drug resistance (MDR) and extensively drug resistance (XDR), as defined in the ReSeqTB database [21], was compared to the clinical DR status of the patient. In cases where the patient was determined to have a DR infection, but the canonical DR SNPs were not present, the variant call format (vcf) file for that sample was examined for evidence of low-frequency DR SNPs. The software package LoFreq was used to determine if statistically significant low frequency variation was present in the raw genomic data [22].

### Phylogenetic analysis

Phylogenetic trees were constructed from single-nucleotide polymorphisms (SNPs) extracted from full-length genomes (16,815 SNPs) and genomes with the PE/PPE loci (as defined by Fishbein et al. [23]) excluded (13,321 SNPs). Phylogenies were calculated using the neighbor-joining algorithm [24] on Hamming distances as implemented in the MEGA7 software [25] and Bayesian analysis using the general time-reversible (GTR) substitution model with gamma-distributed rate variation among sites as implemented in the MrBayes v3.2.5 software [26]. No significant differences were found among the phylogenies calculated using these two algorithms and the two data sets. Unless otherwise specified results are presented for the SNP data exclusive of the PE/PPE protein loci. Network analysis was conducted using the TCS software, version 1.21 [27]. A strict limit of 20 steps was used to infer the network. The presence of SNP loci with unreadable calls was found to distort the counts of pairwise distances. For this reason, further analysis using pairwise distances, including the network analysis, used a genomic SNP array with the indeterminant sites removed (final length of data: 11,202 SNPs).

## Results

**Fig. 1:**
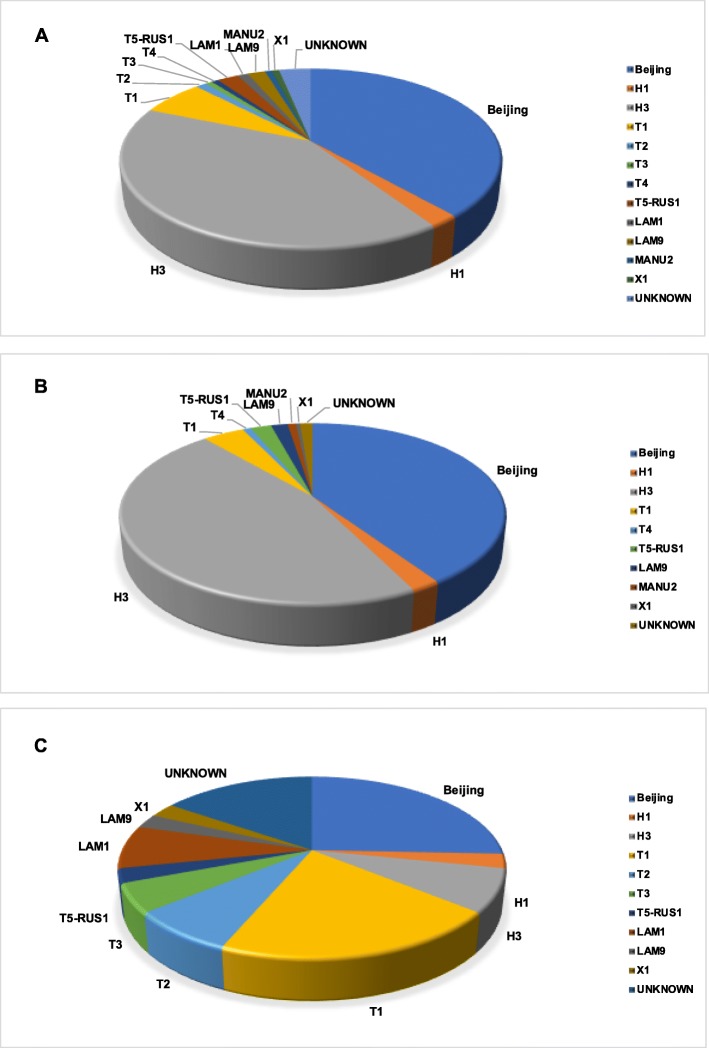
**a** Pie chart of the distribution of spoligotypes among the 278 *Mtb* samples from Moldova. **b** Pie chart of the distribution of spoligotypes among the 239 drug-resistant *Mtb* samples from Moldova. **c** Pie chart of the distribution of spoligotypes among the 39 drug-sensitive *Mtb* samples from Moldova. The category “Unknown” includes spoligotypes that were intermediate to two known spoligotypes as well as novel unclassified spoligotypes

We performed genomic analysis on 278 *Mtb* isolates (239 drug-resistant, 39 drug-sensitive, as determined by standard clinical drug susceptibility tests) from 190 TB patients. The majority of the isolates were from two spoligotypes in nearly equal distribution (41% H3 and 38% Beijing). All H3 spoligotypes had the 4.2.1 numeric SNP barcode and all Beijing spoligotypes had the 2.2.1 numeric SNP barcode. Not all 4.2.1 lineage samples had the H3 spoligotype. The remaining samples were distributed among nine other spoligotypes, all present at a frequency of less than 10% (Fig. 1a). An extremely similar spoligotype distribution, with the H3/4.2.1 and Beijing/2.2.1 spoligotypes being dominant, is seen in the drug-resistant samples (Fig. 1b). There is much more uniform spoligotype variation among drug-sensitive samples (Fig. 1c).

**Fig. 2:**
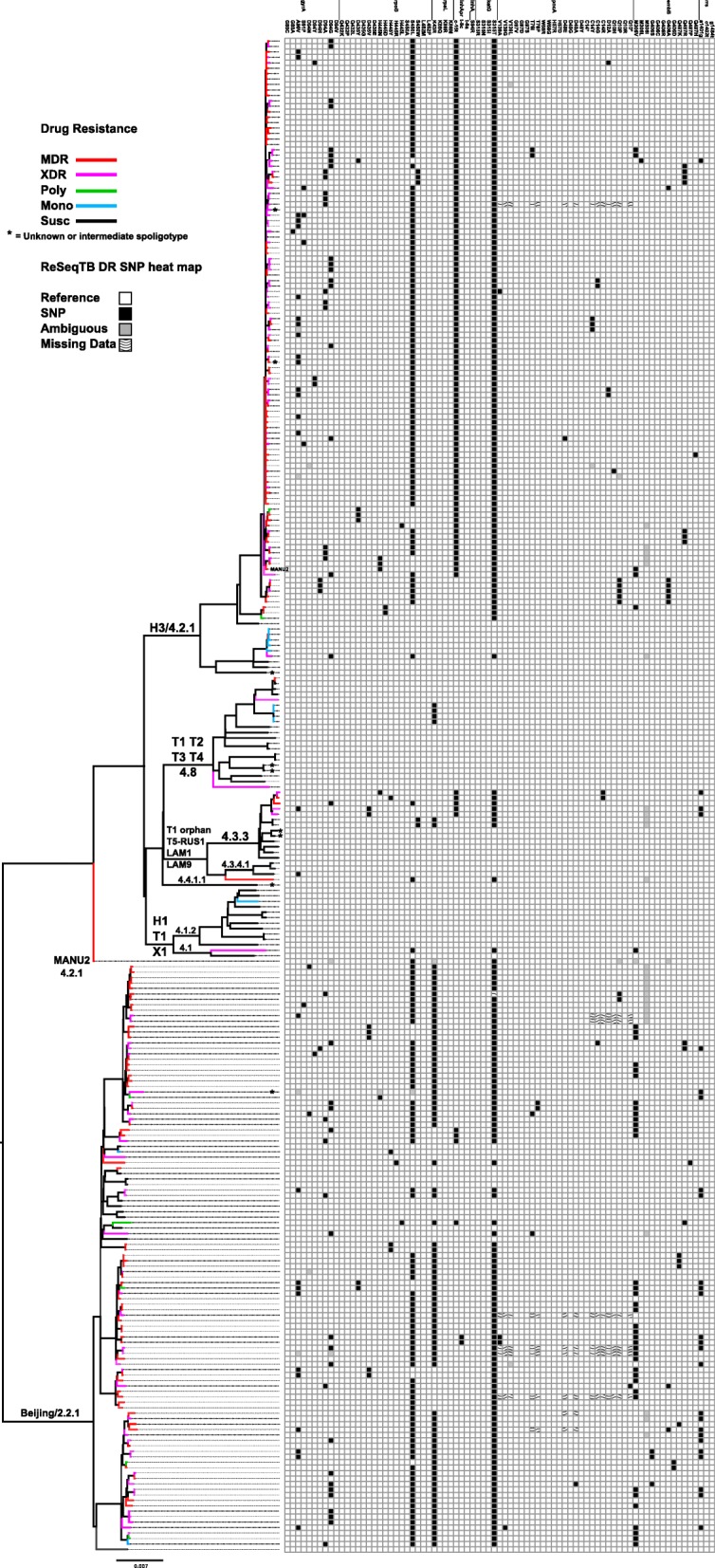
Bayesian phylogenetic tree of 278 Moldovan *Mtb* genomes with heat map of drug resistance SNPs. The Bayesian phylogeny was calculated using 13,321 genomic SNPs. SNPs at PE/PPE protein loci were not included in this calculation. Thick branches have posterior probabilities greater than 0.95. Tree tip branch colors represent drug resistance testing results for each sample, with the result status color indicated in the legend. Spoligotype lineages are labeled at the most ancestral branch for groups belonging to those lineages. The two MANU2 samples did not group together. The heat map shows the presence or absence of drug-resistance SNPs that were determined to be significantly associated with resistance to specific drugs by ReSeqTB. Black cells represent the presence of a DR SNP. Grey cells represent ambiguous calls for a DR SNP. Cells with a wavy fill did not have reliable nucleotide calls at the DR loci

Bayesian phylogenetic analysis of genomic SNPs demonstrated differing evolutionary structures between Beijing/2.2.1 and H3/4.2.1 samples (Fig. 2). The majority of the H3/4.2.1 samples clustered into one shallow, strongly supported (clade posterior probability > 0.95) group (mean Hamming distance 17.1 SNPs, range 0–40 SNPs). Beijing/2.2.1 samples mostly clustered into four well-supported (*P* > 0.95) clades (Fig. 2) which were each more divergent than the main H3/4.2.1 clade (mean pairwise Hamming distances 40.2 (range 0–53), 30.0 (0–53), 35.3 (3–68), and 33.1 (21–31) SNPs). All H3/4.2.1 samples had a smaller mean pairwise diversity (mean Hamming distance 74.4 SNPs) than the Beijing/2.2.1 samples (mean Hamming distance 144.5 SNPs) but a greater range of individual pairwise diversity (Hamming distance range 0–495 for H3/4.2.1, 0–233 for Beijing/2.2.1).

Whole genome variant analysis (WGVA) was performed to reveal molecular patterns of drug resistance within our sample set. The presence or absence of drug-resistance SNPs, as determined by data in the ReSeqTB database (Table 1) [21], were mapped onto the tips of this phylogeny and are presented as a heat map to the right of the tree in Fig. 2. Our analysis shows the rpoB S450 L (rifampicin resistance), inhA promoter c(− 15)t (isoniazid resistance), and katG S315 T (isoniazid resistance) mutations broadly distributed across DR samples in both the H3/4.2.1 and Beijing/2.2.1 clades. The isolates in the H3/4.2.1 clade do not appear to have any mutations in the 16S ribosomal RNA gene *rrs* that would lead to resistance to second-line injectable drugs (amikacin, kanamycin, or capreomycin). Several widely phylogenetically distributed Beijing/2.2.1 samples have the *rrs* a1401g mutation. This distribution implies the *rrs* a1401g mutation has arisen independently multiple times among the divergent Beijing/2.2.1 isolates.

**Table 1:** Drug resistance mutations from ReSeqTB, August 2017. Sensitivity, specificity, and *p*-values for these mutations were calculated by ReSeqTB based on evidence from the literature

DRUG	GENE	MUTATION	SENSITIVITY	SPECIFICITY	*P*-VALUE
RIFAMPICIN	rpoB	Q432K	0.00	1.00	0.01
	rpoB	Q432P	0.00	1.00	0.01
	rpoB	D435Y	0.02	1.00	0.00
	rpoB	D435G	0.00	1.00	0.01
	rpoB	D435V	0.07	1.00	0.00
	rpoB	H445D	0.05	1.00	0.00
	rpoB	H445Y	0.04	1.00	0.00
	rpoB	H445R	0.02	1.00	0.00
	rpoB	S450W	0.01	1.00	0.00
	rpoB	S450L	0.69	1.00	0.00
	rpoB	L452P	0.02	1.00	0.00
ISONIAZID	inhA	S94A	0.01	1.00	0.00
	inhA-Pro	c-15t	0.15	0.99	0.00
	inhA-Pro	t-8a	0.00	1.00	0.01
	inhA-Pro	t-8c	0.01	1.00	0.00
	katG	S315N	0.01	1.00	0.00
	katG	S315T	0.70	0.99	0.00
OFLOXACIN	gyrA	G88C	0.01	1.00	0.04
	gyrA	A90V	0.20	0.99	0.00
	gyrA	S91P	0.07	1.00	0.00
	gyrA	D94N	0.04	1.00	0.00
	gyrA	D94H	0.02	1.00	0.00
	gyrA	D94Y	0.06	1.00	0.00
	gyrA	D94A	0.09	1.00	0.00
	gyrA	D94G	0.34	0.99	0.00
KANAMYCIN	rrs	a1401g	0.57	0.99	0.00
AMIKACIN	rrs	a1401g	0.76	0.99	0.00
	rrs	g1484t	0.01	1.00	0.04
CAPREOMYCIN	rrs	a1401g	0.71	0.96	0.00
	rrs	g1484t	0.01	1.00	0.02
STREPTOMYCIN	rpsL	K43R	0.63	0.99	0.00
	rpsL	K88R	0.05	1.00	0.00
PYRAZINAMIDE	pncA	D49G	0.02	1.00	0.00
	pncA	H57D	0.07	1.00	0.00
	pncA	H57R	0.01	1.00	0.01
	pncA	W68R	0.02	1.00	0.00
	pncA	T76P	0.03	1.00	0.02
	pncA	T76P	0.01	1.00	0.03
	pncA	G97D	0.01	1.00	0.00
	pncA	Q10P	0.13	1.00	0.00
	pncA	D12A	0.01	1.00	0.01
	pncA	C14R	0.01	1.00	0.00
	pncA	V139L	0.01	1.00	0.01
ETHAMBUTOL	embB	G406S	0.01	1.00	0.00
	embB	G406C	0.00	1.00	0.01
	embB	G406D	0.03	1.00	0.00
	embB	G406A	0.02	1.00	0.00
	embB	Q497R	0.10	1.00	0.00
	embB	M306L	0.02	1.00	0.00
	embB	M306V	0.34	0.98	0.00
	embB	M306I	0.23	0.98	0.00
	embB	M306I	0.05	0.99	0.00
	embB	M306I	0.02	1.00	0.00

Susceptibility to first and second-line drugs was determined using phenotypic drug susceptibility testing (pDST) assays. Standard LPAs of drug resistance (Hain MTBDRplus and GenoType MTBDRsl and Cepheid GeneXpert) also were performed on many of these samples. All samples with a LPA also had a phenotypic test, and no LPA results were more severe than phenotypic DST results. There were 12 samples in which pDST-determined drug resistance did not match what was predicted by WGVA (Table 2). In 10 of these cases the WGVA drug resistance profile was “susceptible” while that from pDST was mono-resistant, MDR, or XDR. Two patients with drug susceptible *Mtb* infection, as determined by pDST, had WGVA profiles concordant with mono-resistance.

**Table 2:** Drug resistance phenotype/genotype mismatches

Patient ID	Sample ID	pDST Profile	WGVA Prediction	Has Low Frequency Variation	Mean Coverage < 100 Reads
552	SRR3743495	MDR	Sens	TRUE	
725	SRR5153884	MDR	Sens		
740	SRR5153913	MDR	Sens	TRUE	
764	SRR3743493	Mono^a^	Sens	TRUE	
773	SRR3743482	Mono^a^	Sens	TRUE	
1242	SRR5153868	Sens	rpsL K43R streptomycin resistant		
1247	SRR5153880	Mono^a^	Sens		
1247	SRR5153881	Mono^a^	Sens		
1248	SRR5153901	Sens	gyrA A90V fluoroquinolone resistant		
1259	SRR3743371	MDR	Sens		TRUE
1612	SRR6807669	Mono^a^	Sens		TRUE
1612	SRR6807670	Mono^a^	Sens		TRUE

^a^Streptomycin resistant

Seven of these samples were from paired samples. In two cases (Patient IDs 1247 and 1612) both samples in the pair exhibited genotype/phenotype mismatches. For the other three samples in all cases that later isolate had the genotype/phenotype mismatch. For Patient ID 1248 the paired samples had different spoligotypes while for the other two (Patient IDs 740 and 1242) the spoligotypes were the same.

This discrepancy between pDST and WGVA results could be an indication of mixed infection when there is evidence of low-level DR variants. For the 10 samples with a WGVA drug susceptible profile and a pDST resistant profile, the full genomic read data were examined to determine if low-frequency variants were present at the ReSeqTB DR SNP sites. Analysis with LoFreq [22] found no statistically significant low-frequency variation in these samples. Four of these samples (SRR3743495, SRR5153913, SRR3743493, and SRR3743482) had low level variation (greater than 1% of reads but not statistically significant) at several ReSeqTB DR loci.

The sample with the most low-level variation was SRR5153913 (Patient ID 740). This sample was classified as MDR by the BACTEC and Hain DSTs but it had no SNP calls at ReSeqTB DR SNP sites. This sample had low-frequency variation greater than 1% of reads at rpoB S450 L (4/107 reads), katG S315 T (4/142), inhH promoter c(− 15)t (4/158), rpsL K88R(4/195), and pncA G97D (2/139). These variants would allow subpopulations of the pathogen to grow in cultures containing rifampicin, isoniazid, streptomycin, and pyrazinamide (respectively), corresponding to the MDR profile.

We also examined depth of coverage as a possible explanation for the discrepancy between pDST and WGVA results. Three of the 10 samples with WGVA susceptible/pDST resistant profiles had low coverage (average number < 100 reads) across all of the ReSeqTB DR SNP sites. Due to sampling effects low coverage at DR SNP sites could lead to under-reporting of low-frequency DR SNPs that may be present in the patient. The low frequency variation found at ReSeqTB DR SNP sites in these three samples was on the order of 1% of reads or less.

Two samples (patient/isolate IDs 1242/SRR5153868 and 1248/SRR5153901) were identified as being WGVA-resistant and pDST sensitive (Table 2). Isolate SRR5153868 had the rpsL K43R variant for streptomycin resistance and isolate SRR5153901 had the gyrA A90V variant for ofloxacin resistance. Additionally, SRR5153868 had three DR variants present at very low frequency: rpoB L452P (2/177), pncA Q10P (2/159), and pncA H57D (2/192). Isolate SRR5153901 had one low frequency variant in the rpoB RRDR that was not a recognized DR variant. There are several potential sources for this disagreement, including differences in diversity between WGVA samples and pDST samples due to mixed infection in the original samples [28] and localized failure of individual DSTs.

### Longitudinal sampling

**Fig. 3:**
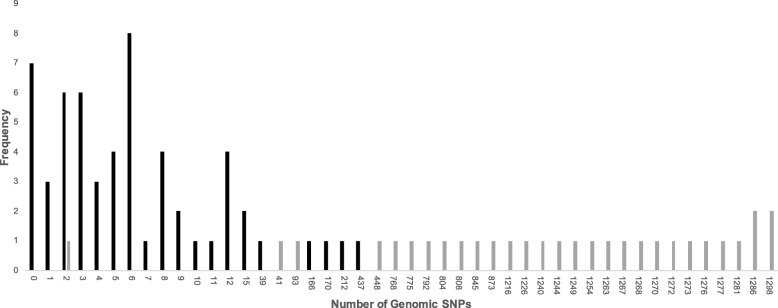
Frequency histogram of pairwise Hamming distance within paired samples. Paired samples with identical lineages (black bars) were analyzed separately from samples with different lineages (grey bars)

Eighty-nine patients had two samples collected at different times (Additional file 1: Table S1). The time span of the paired samples ranged from 46 days to 7.38 years. Forty-six pairs had fewer than 10 SNPs different between their genomes (range 0–9); 7 of those pairs contained identical genomes (Fig. 3). Thirty pairs had different spoligotype lineages, and 27 of these differed by more than 400 SNPs (range: 448–1298).

Five sample pairs had the same spoligotype and SNP barcode lineage within the pair but differed by an elevated number of SNPs (patients 559, 1250, 1605, 1611, and 1614, range 39–437 SNPs), where elevated is defined as greater than 15 SNPs as seen in the break in Fig. 3. The second sample for these patients were labelled “relapse” (except in the case where a patient died and was labelled “failure”). However, in all of these cases the second sample is most likely a reinfection due to the elevated number of SNPs between the two samples. Also, these pairs of samples typically clustered into separate subclades within their main clades, further supporting that they represent multiple infections by independent strains within the H3/4.2.1 or Beijing/2.2.1 spoligotypes.

**Fig. 4:**
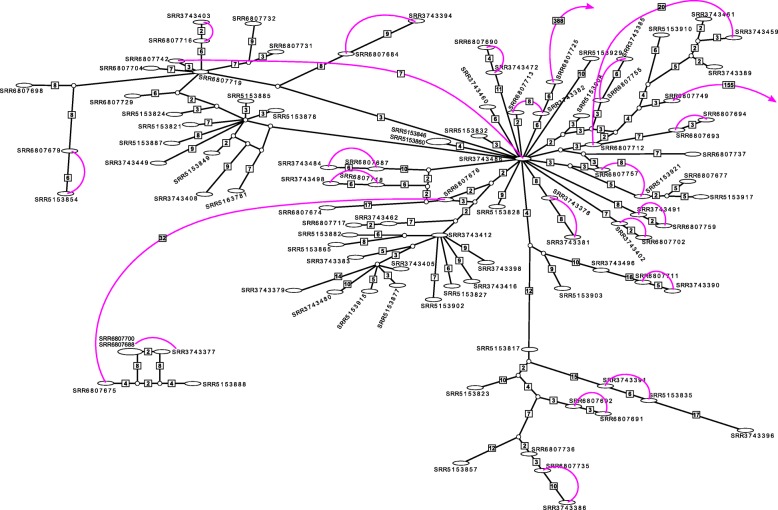
SNP distance network of the H3/4.2.1 clade with a hard limit of 20 SNPs difference. Ovals are samples included in this study. Identical samples are contained in one node, with the node dimensions proportional to the number of identical samples in that node. Circles represent unsampled genotypes intermediate between existing samples. Connections are not to scale to enable legibility. Unlabeled connections indicate a difference of one SNP. Larger numbers of differences are indicated in boxes. Colored connections indicate paired samples. Samples with a paired component not in the H3/4.2.1 clade end with an arrow. Paired samples that are not neighbors in the network have their pairwise SNP difference indicated in a box

We found instances in which samples from other patients were more similar to one of the paired samples than the paired samples were to each other (Fig. 4). These pairs and closely-related samples were all in the H3/4.2.1 clade. This clade contains a large subgroup of samples too similar for reliable determination of phylogenetic relationships, as would be expected for a population of very closely related clonal lineages. To accommodate these ambiguities we performed a network analysis. We found two significant networks: one small network of five samples (two were identical) and a second much larger network of 61 samples (Fig. 4). The larger network was very complex with three main interior nodes from which most other samples were derived. Two of these interior nodes were samples present in the data set (SRR6807719 and SRR3743486) and the third was an unsampled hypothetical genotype. These three nodes were very similar, differing only by two or four SNPs. Only eight of the connections in the 61-sample network were greater than 10 SNPs. Most samples were connected by very few genomic differences, indicating that many of the H3/4.2.1 TB sublineages infecting patients in Moldova are very closely related.

Mapping the paired sample relationships onto these networks showed that most paired samples were each other’s closest relatives, which would be consistent with a possible reactivation in these patients. Even though they had the same spoligotype four paired samples were not each other’s closest relatives, even though they differed by relatively few SNPs (7, 8, 8, and 10 SNPs). While bacterial evolution during drug treatment might account for the minimal SNP differentiation in these four pairs [29], their lack of a direct relationship in the network indicates that these may be cases of reinfection by a closely-related lineage.

## Discussion

We analyzed a range of drug-susceptible and resistant samples from 190 Moldovan TB patients to characterize the local structure of this epidemic. Our analysis presents three main findings: 1) phylogenetic evidence and network analysis suggest a high degree of person-to-person transmission of drug-resistant tuberculosis, 2) one strain is so prevalent that there is extremely little diversity among the patient samples, and 3) low frequency variants in several cases where there is disagreement between phenotypic drug resistance results and whole genomic analysis suggest a mixed infection.

Genomic analysis of local TB outbreaks have typically found one or more circulating strains with an extreme paucity of diversity [20, 30, 31]. These “clones” [20] are interpreted as evidence of rapid person-to-person transmission of a single, typically drug-resistant, lineage rather than the in vivo development of drug resistance over the course of treatment. This pattern of clonality occurs independently of the TB lineages that are present in the local outbreak, consistant with lineage not being a causal factor in this pattern. In Moldova, the H3/4.2.1 samples demonstrated this very shallow phylogenetic structure while the Beijing/2.2.1 samples had a more complex and deeper structure. These patterns are consistent with multiple Beijing/2.2.1 sublineages being responsible for infection in Moldova over the sampling period, while one main H3/4.2.1 sublineage predominated during this time. There were three smaller groups within the Beijing/2.2.1 samples that also were very shallow and closely related, consistent with limited person-to-person transmission of DR TB.

This shallow, highly unresolved phylogenetic structure in the H3/4.2.1 clade prompted a network analysis of these samples (Fig. 4). Several small, two sample, networks were found, in addition to two larger networks. The smaller of these consisted of five samples (two identical) in a straightforward reticulate network. The larger network was very complex with many reticulate paths among nodes. This pattern is consistent with a small number of closely related H3/4.2.1 strains leading to many of the H3/4.2.1 infections in Moldova. This pattern also makes the determination of reinfection or reactivation in the paired samples difficult to ascertain. For four of these pairs the potential of reinfection would not be obvious, as the length of time between samples and the number of SNPs were consistent with the *Mtb* genome substitution rate under selection pressure from antibiotic therapy [29]. In the paired samples with relatively fewer genomic changes (but still greater than neutral expectations), it is also possible that increased mutation rate due to selection from drug treatment and host immune pressure could result in more SNPs between samples than expected. Eldholm et al. [29] observed this among longitudinal samples from an infection that began as susceptible to first-line drugs and progressed to XDR.

The circulation of highly interrelated H3/4.2.1 sublineages in Moldova makes the determination of reactivation or reinfection cases problematic [3, 11, 32, 33]. Our network analysis demonstrates that a sample taken later in time, while having the same spoligotype and numeric SNP barcode, can be more closely related to samples from other individuals than the initial sample from the same patient. Conversely, paired samples with the same spoligotype and numeric SNP barcode but a large number of genomic SNPs between the samples are also evidence of reinfection that was previously classified as “relapse”. Together, our observations of high genomic similarity between circulating strains and large genomic variation between paired samples with the same lineage classification are evidence that person-to-person transmission of DR TB is probably much higher than currently recognized. To a limited extent similar patterns were found in Beijing/2.2.1 sublineages as well, indicating that the effect of circulating strain genealogical structure is not lineage specific.

A portion of samples exhibited discrepancy in drug resistance determination between pDST vs WGVA results, most having “susceptible” results by WGVA despite having been determined “resistant” by pDST. This discrepancy could be due to technical or biological causes. In four cases, low-frequency variants were found by WGVA at DR sites, though this variation was not statistically significant by LoFreq [22] analysis. Depth of coverage could also influence whether low-frequency DR variants were able to be detected at all in our analysis, as a portion of discordant samples had low coverage at DR SNP sites. It is also possible that drug resistance in these samples may be conferred by not-yet identified loci or variants. For example, several pDST-determined XDR samples do not carry SNPs at the *rrs* locus, so resistance may be due to secondary loci which have not been identified as having a strongly significant effect of second-line drug resistance, such as *tlyA* for capreomycin [34] and the *eis* promoter for kanamycin [35]. Our analysis also found low-frequency DR variants in one of the two cases with pDST-sensitive profiles. As these samples had one recognized DR variant each this suggests that drug resistance was not detected by culture-based methods due to growth and/or selection conditions. Finally, mixed infections could also lead to the pattern of lineage divergence found among the longitudinal samples. If treatment removes the susceptible majority lineage, subsequent samples could primarily consist of the minority lineage which would appear to be reinfection but would actually be a persistent infection.

## Conclusions

Our study sought to better understand the impact of genomic diversity, evolution, and epidemiology of DR-TB on the ability to determine recurrence status of infections. The nature of the current DR TB outbreak in Moldova was well-suited for this analysis. Whole genome sequencing provided detailed information about the population structure of local circulating strains and the variety of strains infecting individual patients. Genomic sequencing provided strong evidence of a widespread clonal strain with very low diversity among these samples. For this population structure it is just as likely that individuals are being reinfected by a circulating closely related strain as it is that their initial infection was not entirely eradicated by treatment. Genomic sequencing also provided strong evidence that cases previously classified as relapse (based on locus-specific genotyping) were, in fact, reinfection due to the large genomic diversity between the paired longitudinal samples. While specific to our samples, these results provide some actionable insights into local TB epidemics and ways to control them. As more evidence accumulates that a significant portion of existing cases of DR TB are the result of reinfection, either by very similar or divergent (but having the same classification) strains, additional efforts must be put into reducing transmission, especially person-to-person transmission in clinical settings. Our results showing mixed infection in some patients demonstrates the importance of both phenotypic and genotypic methods for TB diagnosis and drug resistance testing. Genomic sequencing at greater depth will provide stronger evidence of existing low-level mixed infections. These data will give us a better understanding of the frequency of mixed TB infection and its impact on TB outcomes.

## Supplementary information

**Additional file 1: Table S1.** File containing data about paired samples analyzed in this article. These data include the SRA identifiers, TB Portal anonymized patient identifier, octal spoligotype, spoligotype and SNP barcode numerical lineage, pairwise distance (number of SNPs) between samples, days between sample collection, maximum drug resistance test result, case definition, case outcome, patient age at onset, and patient gender. A key to abbreviations used in the table are included in the header.

## Data Availability

The datasets supporting the conclusions of this article are available in the NCBI Short Read Archive under Bioprojects PRJNA436997 and PRJNA318002.

## Abbreviations

DR-TB: Drug-resistant tuberculosis
LPA: Line probe assay
MDR: Multidrug-resistant
pDST: Phenotypic drug susceptibility testing
TB: Tuberculosis
WGVA: Whole-genome variant analysis
XDR: Extensively drug-resistant
