# Supplementary material for: A retrospective genomic analysis of drug-resistant strains of M. tuberculosis in a high-burden setting, with an emphasis on comparative diagnostics and reactivation and reinfection status
Source: BMC Infect Dis. 2020 Jan 7;20:17. doi: 10.1186/s12879-019-4739-z (PMC6947865; doi:10.1186/s12879-019-4739-z)
Supplement: Supplementary file 1 — Additional file 1: Table S1. File containing data about paired samples analyzed in this article. These data include the SRA identifiers, TB Portal anonymized patient identifier, octal spoligotype, spoligotype and SNP barcode numerical lineage, pairwise distance (number of SNPs) between samples, days between sample collection, maximum drug resistance test result, case definition, case outcome, patient age at onset, and patient gender. A key to abbreviations used in the table are included in the header. [file 12879_2019_4739_MOESM1_ESM.docx]

Paired samples from Moldova. “nr” = not reported. Case Definition: F = Failure, N = New, O = Other, R = Relapse, L = Lost to follow-up. Case Outcome: C = Cured, D = Died, Df = Default, F = Failure, S = Still on treatment.

| **NCBI SRA ID** | **TB Portal Patient Identifier** | **Octal Spoligotype** | **Lineage** | **Pairwise distance (Number of SNPs)** | **Days Between Specimens** | **Drug Resistance, Maximum Test Result** | **Case Definition** | **Case Outcome** | **Age at Onset** | **Gender** |
| --- | --- | --- | --- | --- | --- | --- | --- | --- | --- | --- |
| SRR6807718 | 502 | 774777777420771 | H3/4.2.1 |  |  | MDR | N | F | 29 | Male |
| SRR3743498 | 502 | 774777777420771 | H3/4.2.1 | 6 | 1932 | MDR | F | S | 35 | Male |
| SRR6807688 | 505 | 774777777420771 | H3/4.2.1 |  |  | MDR | L | D | 56 | Male |
| SRR3743377 | 505 | 774777777420771 | H3/4.2.1 | 2 | 46 | MDR | R | D | 56 | Male |
| SRR6807762 | 508 | 000000000003771 | Beijing/2.2.1 |  |  | MDR | N | Df | 27 | Female |
| SRR3743386 | 508 | 774777777420771 | H3/4.2.1 | 1272 | 2154 | XDR | L | C | 33 | Female |
| SRR6807687 | 515 | 774777777420771 | H3/4.2.1 |  |  | MDR | F | F | 33 | Male |
| SRR3743484 | 515 | 774777777420771 | H3/4.2.1 | 6 | 1158 | XDR | F | D | 36 | Male |
| SRR6807709 | 534 | 000000000003771 | Beijing/2.2.1 |  |  | MDR | R | F | 37 | Male |
| SRR3743409 | 534 | 000000000003771 | Beijing/2.2.1 | 8 | 1907 | XDR | F | S | 42 | Male |
| SRR6807705 | 539 | 000000000003771 | Beijing/2.2.1 |  |  | MDR | N | F | 45 | Male |
| SRR3743489 | 539 | 000000000003771 | Beijing/2.2.1 | 1 | 859 | XDR | F | F | 47 | Male |
| SRR6807700 | 541 | 774777777420771 | H3/4.2.1 |  |  | MDR | N | D | 28 | Male |
| SRR3743400 | 541 | 000000000003771 | Beijing/2.2.1 | 1263 | 211 | MDR | F | D | 29 | Male |
| SRR6807708 | 542 | 000000000003771 | Beijing/2.2.1 |  |  | MDR | R | C | 24 | Male |
| SRR3743494 | 542 | 000000000003771 | Beijing/2.2.1 | 6 | 687 | MDR | F | C | 26 | Male |
| SRR6807707 | 543 | 000000000003771 | Beijing/2.2.1 |  |  | MDR | N | F | 32 | Male |
| SRR3743475 | 543 | 000000000003771 | Beijing/2.2.1 | 4 | 1175 | XDR | F | C | 35 | Male |
| SRR6807761 | 544 | 773777777420771 | T1/4.8 |  |  | Sens | N | C | 33 | Male |
| SRR3743380 | 544 | 777777607660771 | LAM9/4.3.3 | 775 | 2175 | MDR | F | D | 39 | Male |
| SRR6807755 | 546 | 774777777420771 | H3/4.2.1 |  |  | MDR | N | C | 39 | Male |
| SRR3743385 | 546 | 774777777420771 | H3/4.2.1 | 6 | 420 | XDR | F | C | 40 | Male |
| SRR6807756 | 548 | 000000000003771 | Beijing/2.2.1 |  |  | MDR | N | Df | 36 | Male |
| SRR3743499 | 548 | 000000000003771 | Beijing/2.2.1 | 6 | 768 | MDR | O | Df | 38 | Male |
| SRR6807763 | 551 | 000000000003771 | Beijing/2.2.1 |  |  | Poly | R | D | 61 | Male |
| SRR3743492 | 551 | 000000000003771 | Beijing/2.2.1 | 15 | 1594 | MDR | N | D | 66 | Male |
| SRR6807743 | 559 | 000000000003771 | Beijing/2.2.1 |  |  | Sens | nr | D | 47 | Male |
| SRR3743487 | 559 | 000000000003771 | Beijing/2.2.1 | 212 | 2172 | XDR | F | D | 53 | Male |
| SRR6807684 | 580 | 774777777420771 | H3/4.2.1 |  |  | MDR | F | F | 29 | Female |
| SRR3743394 | 580 | 774777777420771 | H3/4.2.1 | 9 | 1331 | XDR | R | D | 33 | Female |
| SRR6807681 | 581 | 000000000003771 | Beijing/2.2.1 |  |  | MDR | R | F | 57 | Male |
| SRR3743373 | 581 | 000000000003771 | Beijing/2.2.1 | 12 | 616 | XDR | R | S | 59 | Male |
| SRR6807686 | 583 | 000000000003771 | Beijing/2.2.1 |  |  | XDR | F | F | 49 | Male |
| SRR3743375 | 583 | 000000000003771 | Beijing/2.2.1 | 8 | 2009 | XDR | R | D | 54 | Male |
| SRR6807680 | 584 | 777777777760741 | T1/4.8 |  |  | Sens | R | D | 42 | Male |
| SRR3743397 | 584 | 000000000003771 | Beijing/2.2.1 | 1298 | 1996 | XDR | L | D | 48 | Male |
| SRR6807689 | 588 | 774777777420771 | H3/4.2.1 |  |  | MDR | N | C | 54 | Male |
| SRR3743437 | 588 | 774777777420771 | H3/4.2.1 | 3 | 2393 | XDR | F | C | 59 | Male |
| SRR5153835 | 599 | 774777777420771 | H3/4.2.1 |  |  | XDR | R | C | 26 | Female |
| SRR3743391 | 599 | 774777777420771 | H3/4.2.1 | 6 | 971 | XDR | R | C | 29 | Female |
| SRR6807715 | 600 | 000000000003771 | Beijing/2.2.1 |  |  | MDR | R | D | 40 | Male |
| SRR3743479 | 600 | 774777777420771 | H3/4.2.1 | 1267 | 2338 | XDR | L | D | 46 | Male |
| SRR6807716 | 602 | 774777777420771 | H3/4.2.1 |  |  | XDR | N | D | 53 | Male |
| SRR3743403 | 602 | 774777777420771 | H3/4.2.1 | 2 | 1030 | MDR | R | D | 56 | Male |
| SRR6807713 | 603 | 774777777420771 | H3/4.2.1 |  |  | XDR | N | F | 35 | Male |
| SRR3743382 | 603 | 774777777420771 | H3/4.2.1 | 10 | 1217 | MDR | F | S | 39 | Male |
| SRR6807711 | 613 | 774777777420771 | H3/4.2.1 |  |  | MDR | R | C | 33 | Male |
| SRR3743390 | 613 | 774737777420771 | H3/4.2.1 | 5 | 1177 | XDR | F | C | 36 | Male |
| SRR6807712 | 627 | 774777777420771 | H3/4.2.1 |  |  | XDR | R | F | 35 | Male |
| SRR3743459 | 627 | 774777777420771 | H3/4.2.1 | 15 | 1843 | MDR | F | D | 40 | Male |
| SRR6807717 | 630 | 774777777421771 | MANU2,H3/4.2.1 |  |  | MDR | N | F | 40 | Male |
| SRR3743462 | 630 | 774777777420771 | H3/4.2.1 | 2 | 1237 | XDR | F | S | 43 | Male |
| SRR6807706 | 632 | 000000000003771 | Beijing/2.2.1 |  |  | MDR | R | F | 45 | Male |
| SRR3743370 | 632 | 000000000003771 | Beijing/2.2.1 | 9 | 604 | MDR | F | D | 47 | Male |
| SRR3743378 | 635 | 774777777420771 | H3/4.2.1 |  |  | XDR | L | C | 33 | Male |
| SRR3743381 | 635 | 774777777420771 | H3/4.2.1 | 8 | 300 | XDR | L | C | 33 | Male |
| SRR6807699 | 640 | 000000000003771 | Beijing/2.2.1 |  |  | MDR | N | F | 27 | Female |
| SRR3743480 | 640 | 774777777420771 | H3/4.2.1 | 1276 | 1322 | XDR | F | C | 30 | Female |
| SRR6807702 | 641 | 774777777420771 | H3/4.2.1 |  |  | MDR | N | D | 33 | Female |
| SRR3743402 | 641 | 774777777420771 | H3/4.2.1 | 2 | 847 | XDR | L | D | 36 | Female |
| SRR6807758 | 645 | 000000000003771 | Beijing/2.2.1 |  |  | MDR | R | C | 20 | Male |
| SRR3743392 | 645 | 000000000003771 | Beijing/2.2.1 | 12 | 1183 | XDR | R | S | 23 | Male |
| SRR6807760 | 646 | 000000000003771 | Beijing/2.2.1 |  |  | MDR | R | D | 51 | Female |
| SRR3743407 | 646 | 000000000003771 | Beijing/2.2.1 | 1 | 1169 | XDR | F | D | 54 | Female |
| SRR6807754 | 648 | 777760003760771 | T5-RUS1/4.3.3 |  |  | MDR | R | D | 29 | Male |
| SRR3743476 | 648 | 777760003760771 | T5-RUS1/4.3.3 | 11 | 1567 | XDR | F | D | 34 | Male |
| SRR5153930 | 651 | 000000000003771 | Beijing/2.2.1 |  |  | XDR | R | Df | 50 | Male |
| SRR3743500 | 651 | 000000000003771 | Beijing/2.2.1 | 7 | 1023 | XDR | R | C | 53 | Male |
| SRR6807742 | 652 | 774777777420771 | H3/4.2.1 |  |  | MDR | R | D | 32 | Male |
| SRR3743486 | 652 | 774777777420771 | H3/4.2.1 | 8 | 943 | MDR | F | D | 34 | Male |
| SRR6807739 | 653 | 000000000003771 | Beijing/2.2.1 |  |  | Poly | N | F | 25 | Male |
| SRR3743376 | 653 | 770012634003771 | UNKNOWN4.2.1 | 41 | 989 | MDR | F | S | 28 | Male |
| SRR6807682 | 698 | 000000000003771 | Beijing/2.2.1 |  |  | MDR | N | D | 41 | Male |
| SRR5153828 | 698 | 774777777422771 | MANU2,H3/4.2.1 | 1270 | 875 | MDR | L | D | 43 | Male |
| SRR5153839 | 702 | 777760007760771 | T5-RUS1 |  |  | MDR | R | D | 65 | Male |
| SRR5153844 | 702 | 777760007760771 | T5-RUS1 | 1 | 2267 | MDR | R | D | 71 | Male |
| SRR6807714 | 707 | 774777777420771 | H3/4.2.1 |  |  | MDR | R | Df | 46 | Male |
| SRR5153838 | 707 | 774777777420771 | H3/4.2.1 | 6 | 2086 | MDR | F | C | 51 | Male |
| SRR5153846 | 714 | 774777777420771 | H3/4.2.1 |  |  | MDR | R | C | 34 | Male |
| SRR5153860 | 714 | 774777777420771 | H3/4.2.1 | 0 | 2491 | MDR | R | S | 40 | Male |
| SRR5153866 | 715 | 777777774020771 | H1/4.1.2.1 |  |  | Sens | R | C | 20 | Female |
| SRR5153865 | 715 | 774777777420771 | H3/4.2.1 | 792 | 1008 | MDR | R | C | 23 | Female |
| SRR6807710 | 719 | 000000000003771 | Beijing/2.2.1 |  |  | MDR | N | F | 29 | Male |
| SRR5153855 | 719 | 774777777423771 | MANU2 | 93 | 2331 | MDR | F | S | 35 | Male |
| SRR5153900 | 732 | 000000000003771 | Beijing/2.2.1 |  |  | Sens | N | D | 58 | Male |
| SRR5153906 | 732 | 774777777420771 | H3/4.2.1 | 1244 | 2334 | MDR | R | D | 64 | Male |
| SRR5153911 | 739 | 000000000003771 | Beijing/2.2.1 |  |  | MDR | R | C | 25 | Male |
| SRR5153909 | 739 | 777776777760771 | X1/4.1 | 1216 | 263 | Sens | R | C | 26 | Male |
| SRR5153914 | 740 | 777777777760741 | T1/4.8 |  |  | Sens | N | C | 37 | Male |
| SRR5153913 | 740 | 777777777760741 | T1/4.8 | 3 | 2196 | MDR | R | D | 43 | Male |
| SRR6807690 | 758 | 774777777420771 | H3/4.2.1 |  |  | MDR | R | Df | 42 | Male |
| SRR3743472 | 758 | 774777777420771 | H3/4.2.1 | 4 | 693 | MDR | R | Df | 44 | Male |
| SRR6807759 | 771 | 774777777420771 | H3/4.2.1 |  |  | MDR | N | F | 28 | Male |
| SRR3743491 | 771 | 774777777420771 | H3/4.2.1 | 2 | 1067 | MDR | L | S | 31 | Male |
| SRR5153924 | 777 | 000000000003771 | Beijing/2.2.1 |  |  | MDR | R | C | 45 | Male |
| SRR3743481 | 777 | 000000000003771 | Beijing/2.2.1 | 0 | 384 | MDR | R | S | 46 | Male |
| SRR6807685 | 1238 | 000000000003771 | Beijing/2.2.1 |  |  | MDR | F | Df | 31 | Male |
| SRR5153834 | 1238 | 000000000003771 | Beijing/2.2.1 | 4 | 2674 | XDR | R | D | 38 | Male |
| SRR5153845 | 1241 | 000000000003771 | Beijing/2.2.1 |  |  | Sens | R | C | 18 | Male |
| SRR5153847 | 1241 | 677777607760771 | LAM1/4.3.4.1 | 1281 | 1457 | Sens | R | C | 21 | Male |
| SRR5153869 | 1242 | 777777777760771 | T1/4.8 |  |  | Mono | R | C | 43 | Male |
| SRR5153868 | 1242 | 777777777760771 | T1/4.8 | 0 | 1044 | Sens | R | S | 46 | Male |
| SRR5153867 | 1243 | 777777777760771 | T1/4.8 |  |  | Mono | N | Df | 39 | Male |
| SRR5153842 | 1243 | 777777777760771 | T1/4.8 | 0 | 1823 | Mono | R | S | 44 | Male |
| SRR5153870 | 1244 | 777777777760741 | T1/4.8 |  |  | Sens | L | C | 29 | Male |
| SRR5153861 | 1244 | 777777774020771 | H1/4.1.2.1 | 804 | 1970 | Sens | R | C | 35 | Male |
| SRR5153843 | 1245 | 000000000003771 | Beijing/2.2.1 |  |  | Sens | R | C | 40 | Male |
| SRR5153850 | 1245 | 777777607760771 | LAM9/4.3.3 | 1268 | 2093 | MDR | R | C | 46 | Male |
| SRR5153858 | 1246 | 777777301760771 | T2,T1,LAM10-CAM/4.8 |  |  | Sens | N | C | 51 | Male |
| SRR5153862 | 1246 | 777777301760771 | T2,T1,LAM10-CAM/4.8 | 3 | 1381 | Sens | R | C | 54 | Male |
| SRR5153880 | 1247 | 777737777420771 | H3/4.2.1 |  |  | Mono | R | C | 35 | Male |
| SRR5153881 | 1247 | 777737777420771 | H3/4.2.1 | 0 | 958 | Mono | R | C | 37 | Male |
| SRR5153905 | 1248 | 000000000003771 | Beijing/2.2.1 |  |  | MDR | N | C | 38 | Male |
| SRR5153901 | 1248 | 677777607760771 | LAM1/4.3.4.1 | 1298 | 2124 | Sens | R | C | 44 | Male |
| SRR5153918 | 1249 | 777777777760771 | T1/4.8 |  |  | Sens | R | C | 51 | Male |
| SRR5153922 | 1249 | 777777777760771 | T1/4.8 | 2 | 2269 | Sens | R | C | 57 | Male |
| SRR5153925 | 1250 | 000000000003771 | Beijing/2.2.1 |  |  | MDR | N | Df | 29 | Male |
| SRR5153927 | 1250 | 000000000003771 | Beijing/2.2.1 | 166 | 1948 | Sens | R | Df | 35 | Male |
| SRR5153851 | 1252 | 777773377760771 | T4/4.8 |  |  | Sens | N | C | 50 | Male |
| SRR5153836 | 1252 | 777773377760771 | T4/4.8 | 0 | 1319 | Sens | R | C | 54 | Male |
| SRR6807679 | 1254 | 774777777420771 | H3/4.2.1 |  |  | MDR | N | F | 23 | Male |
| SRR5153854 | 1254 | 774777777420771 | H3/4.2.1 | 6 | 476 | MDR | L | S | 25 | Male |
| SRR6807704 | 1257 | 774777777420771 | H3/4.2.1 |  |  | MDR | R | C | 42 | Male |
| SRR5153879 | 1257 | 000003777420771 | UNKNOWN/4.2.1 | 448 | 1758 | Sens | R | F | 47 | Male |
| SRR6807703 | 1258 | 000000000003771 | Beijing/2.2.1 |  |  | MDR | N | F | 32 | Male |
| SRR5153864 | 1258 | 000000000003771 | Beijing/2.2.1 | 5 | 1403 | XDR | F | S | 36 | Male |
| SRR6807701 | 1260 | 000000000003771 | Beijing/2.2.1 |  |  | MDR | N | F | 28 | Male |
| SRR5153886 | 1260 | 000000000003771 | Beijing/2.2.1 | 12 | 432 | MDR | R | S | 29 | Male |
| SRR6807757 | 1264 | 774777777420771 | H3/4.2.1 |  |  | MDR | N | F | 28 | Male |
| SRR5153921 | 1264 | 774777777420771 | H3/4.2.1 | 8 | 2457 | MDR | L | D | 34 | Male |
| SRR6807734 | 1562 | 677700003760771 | T1/4.8 |  |  | Sens | R | C | 44 | Male |
| SRR6807733 | 1562 | 000000000003771 | Beijing/2.2.1 | 1314 | 1246 | MDR | R | F | 47 | Male |
| SRR6807735 | 1563 | 774777777420771 | H3/4.2.1 |  |  | Poly | R | C | 32 | Male |
| SRR6807736 | 1563 | 774777777420771 | H3/4.2.1 | 3 | 2694 | MDR | R | S | 39 | Male |
| SRR6807738 | 1564 | 777777777760771 | T1/4.8 |  |  | Sens | N | C | 60 | Male |
| SRR6807737 | 1564 | 774777777420771 | H3/4.2.1 | 808 | 1780 | MDR | R | S | 64 | Male |
| SRR6807730 | 1565 | 000000000003771 | Beijing/2.2.1 |  |  | Sens | N | C | 33 | Male |
| SRR6807729 | 1565 | 774777777420771 | H3/4.2.1 | 1273 | 1438 | MDR | R | S | 37 | Male |
| SRR6807722 | 1566 | 760777774020771 | H1/4.1.2.1 |  |  | Sens | N | C | 35 | Male |
| SRR6807721 | 1566 | 000000000003771 | Beijing/2.2.1 | 1240 | 1399 | Poly | N | S | 39 | Male |
| SRR6807719 | 1604 | 774777777420771 | H3/4.2.1 |  |  | MDR | R | C | 35 | Male |
| SRR6807720 | 1604 | 000000000003771 | Beijing/2.2.1 | 1254 | 1082 | MDR | R | S | 38 | Male |
| SRR6807726 | 1605 | 777737777420771 | H3/4.2.1 |  |  | Sens | R | C | 59 | Female |
| SRR6807725 | 1605 | 774777777420771 | H3/4.2.1 | 437 | 784 | MDR | R | S | 61 | Female |
| SRR6807724 | 1606 | 000000000003771 | Beijing/2.2.1 |  |  | MDR | R | C | 23 | Male |
| SRR6807723 | 1606 | 000000000003771 | Beijing/2.2.1 | 5 | 1632 | MDR | R | F | 28 | Male |
| SRR6807728 | 1607 | 777777777760771 | T1/4.8 |  |  | Sens | R | C | 39 | Male |
| SRR6807727 | 1607 | 000000007760771 | LAM3,T1,H3/4.4.1.1 | 768 | 2541 | Sens | R | C | 46 | Male |
| SRR6807674 | 1608 | 774777777420771 | H3/4.2.1 |  |  | MDR | N | C | 40 | Male |
| SRR6807673 | 1608 | 000000000003771 | Beijing/2.2.1 | 1277 | 1096 | MDR | N | S | 43 | Male |
| SRR6807671 | 1609 | 377777774020771 | H1/4.1.2.1 |  |  | Sens | N | C | 40 | Male |
| SRR6807672 | 1609 | 000000000003771 | Beijing/2.2.1 | 1226 | 1831 | MDR | R | S | 45 | Male |
| SRR6807678 | 1610 | 000000000003771 | Beijing/2.2.1 |  |  | Sens | R | C | 39 | Male |
| SRR6807677 | 1610 | 774777777420771 | H3/4.2.1 | 1249 | 834 | MDR | R | D | 41 | Male |
| SRR6807676 | 1611 | 774777777420771 | H3/4.2.1 |  |  | MDR | N | C | 25 | Male |
| SRR6807675 | 1611 | 774777777420771 | H3/4.2.1 | 39 | 1842 | MDR | R | S | 30 | Male |
| SRR6807669 | 1612 | 777737767420771 | H3/4.2.1 |  |  | Mono | N | C | 51 | Male |
| SRR6807670 | 1612 | 777737777420771 | H3/4.2.1 | 12 | 1330 | Mono | R | C | 54 | Male |
| SRR6807747 | 1613 | 000000000003771 | Beijing/2.2.1 |  |  | Mono | N | C | 28 | Female |
| SRR6807746 | 1613 | 774777777420771 | H3/4.2.1 | 1286 | 1147 | MDR | R | S | 32 | Female |
| SRR6807748 | 1614 | 774777777420771 | H3/4.2.1 |  |  | Poly | N | C | 42 | Male |
| SRR6807749 | 1614 | 774777777420771 | H3/4.2.1 | 170 | 1524 | MDR | R | D | 46 | Male |
| SRR6807750 | 1615 | 000000000003771 | Beijing/2.2.1 |  |  | MDR | N | C | 29 | Male |
| SRR6807751 | 1615 | 000000000003771 | Beijing/2.2.1 | 0 | 821 | MDR | R | C | 32 | Male |
| SRR6807752 | 1616 | 777777777760731 | T2/4.8 |  |  | Sens | N | C | 24 | Male |
| SRR6807753 | 1616 | 000000000003771 | Beijing/2.2.1 | 1286 | 2266 | MDR | R | S | 30 | Male |
| SRR6807745 | 1617 | 777737777420771 | H3/4.2.1 |  |  | Sens | R | C | 43 | Female |
| SRR6807744 | 1617 | 777000000000371 | UNKNOWN/4.3.3 | 845 | 672 | Sens | R | C | 45 | Female |
| SRR6807692 | 1618 | 774777777420771 | H3/4.2.1 |  |  | MDR | R | C | 59 | Male |
| SRR6807691 | 1618 | 774777777420771 | H3/4.2.1 | 3 | 1308 | MDR | R | S | 63 | Male |
| SRR6807693 | 1619 | 774777777420771 | H3/4.2.1 |  |  | MDR | R | C | 61 | Male |
| SRR6807694 | 1619 | 774777777420771 | H3/4.2.1 | 3 | 1184 | MDR | R | S | 64 | Male |
| SRR6807696 | 1620 | 000000000003771 | Beijing/2.2.1 |  |  | Poly | R | C | 39 | Male |
| SRR6807695 | 1620 | 000000000003771 | Beijing/2.2.1 | 5 | 1810 | MDR | N | S | 44 | Male |
| SRR6807697 | 1621 | 777777777760771 | T1/4.8 |  |  | Sens | R | C | 52 | Male |
| SRR6807698 | 1621 | 774777777420771 | H3/4.2.1 | 873 | 2088 | MDR | R | S | 57 | Male |
| SRR6807741 | 1622 | 000000000003771 | Beijing/2.2.1 |  |  | MDR | R | C | 54 | Female |
| SRR6807740 | 1622 | 000000000003771 | Beijing/2.2.1 | 2 | 1615 | MDR | R | F | 58 | Female |
